# Using support vector machines on photoplethysmographic signals to discriminate between hypovolemia and euvolemia

**DOI:** 10.1371/journal.pone.0195087

**Published:** 2018-03-29

**Authors:** Natasa Reljin, Gary Zimmer, Yelena Malyuta, Kirk Shelley, Yitzhak Mendelson, David J. Blehar, Chad E. Darling, Ki H. Chon

**Affiliations:** 1 Department of Biomedical Engineering, University of Connecticut, Storrs, CT, United States of America; 2 Campbell University School of Medicine, Buies Creek, NC, United States of America; 3 Department of Emergency Medicine, University of Massachusetts Medical School, Worcester, MA, United States of America; 4 Department of Anesthesiology, Yale University School of Medicine, New Haven, CT, United States of America; 5 Department of Biomedical Engineering, Worcester Polytechnic Institute, Worcester, MA, United States of America; The University of Tokyo, JAPAN

## Abstract

Identifying trauma patients at risk of imminent hemorrhagic shock is a challenging task in intraoperative and battlefield settings given the variability of traditional vital signs, such as heart rate and blood pressure, and their inability to detect blood loss at an early stage. To this end, we acquired *N* = 58 photoplethysmographic (PPG) recordings from both trauma patients with suspected hemorrhage admitted to the hospital, and healthy volunteers subjected to blood withdrawal of 0.9 L. We propose four features to characterize each recording: goodness of fit (*r*^2^), the slope of the trend line, percentage change, and the absolute change between amplitude estimates in the heart rate frequency range at the first and last time points. Also, we propose a machine learning algorithm to distinguish between blood loss and no blood loss. The optimal overall accuracy of discriminating between hypovolemia and euvolemia was 88.38%, while sensitivity and specificity were 88.86% and 87.90%, respectively. In addition, the proposed features and algorithm performed well even when moderate blood volume was withdrawn. The results suggest that the proposed features and algorithm are suitable for the automatic discrimination between hypovolemia and euvolemia, and can be beneficial and applicable in both intraoperative/emergency and combat casualty care.

## Introduction

Traumatic injury is the leading cause of death worldwide, with hemorrhage being accountable for about 40% of mortality [1]. Therefore, accurate assessment of trauma patients for hemorrhage is very important in emergency and operative rooms, as well as on the battlefield. If not properly and promptly treated, massive bleeding, i.e., more than 30% of blood loss, may lead to hemorrhagic shock and death [2]. Humans’ hemodynamic response consists of two phases. During the first phase (arterial baroreceptor-mediated phase), as cardiac output decreases, heart rate (HR) increases, as does the resistance in arteries in order to increase cardiac output and to maintain normal values of arterial blood pressure (BP) [3–5]. As blood loss progresses and blood volume falls to a critical level, which is usually 30% of normal, the second phase develops. Compensatory mechanisms are now overwhelmed by the losses, which leads to decrease in both HR and BP [3–5]. At this time, blood flow to the brain and heart is restricted, thus posing a life-threatening situation. Since routinely measured vital signs such as heart rate and blood pressure vary and do not reflect the symptoms of blood loss until at least 30% of circulating blood volume is lost [2], there exists a need for automatic blood loss detection that can be used in both emergency/critical care and combat casualty care.

To this end photoplethysmographic (PPG) signals found applications in blood loss detection. PPG signals are recorded by pulse oximeter (PO), a non-invasive and easy-to-use device that has been commonly employed tool to monitor HR and arterial oxygen saturation (S_p_O_2_). Recently, several studies have proposed the use of PPG signals to detect blood loss by characterizing them using various features (respiration-induced variation in PPG at respiratory frequency; pulse amplitude, width and area under the curve from PPG signals; instantaneous amplitude modulations in heart rate and breathing rate frequency ranges; features describing the amplitude of PPG signal; trend of the estimated amplitudes from the HR frequency range from PPG signal) [6–11].

Accurate detection of blood loss is a frequent topic in the literature. Determining and understanding cardiovascular responses to acute hemorrhage is a very important and challenging task. Since enrollment of subjects with traumatic hemorrhage is not easy to perform, researchers have been exploring various models to simulate blood loss. One such model is lower body negative pressure (LBNP). McGrath and co-workers explored whether features of the PPG (pulse amplitude, width, and area under the curve) obtained from three locations (ear, finger, and forehead) could track progressive reductions in blood volume induced by LBNP, and whether their changes could provide an indication of blood loss before changes in arterial blood pressure [7]. All three features were decreasing in all PPG waveforms with the increase of LBNP, while changes in PPG features were observed prior to decrease in blood pressure (at 60% of LBNP tolerance). In one of the previous works performed in our lab, photoplethysmographic waveforms from ear, finger and forehead locations were analyzed with a high-resolution time-frequency spectral (TFS) technique in spontaneously breathing healthy subjects [8]. Two features were procured from the high-resolution TFS: the instantaneous amplitude modulations in heart rate and breathing rate frequency ranges. The results implied that amplitude changes in both frequency ranges, especially in the heart rate frequency band, can be used for early detection (as early as 20% LBNP tolerance) of blood loss when none of the vital signs showed such significant changes. Convertino and co-workers estimated a compensatory reserve index (CRI) from healthy volunteers under LBNP, and obtained a linear relationship between changes in arterial waveforms and compensatory mechanisms [12].

Even though LBNP represents a good model for studying acute hemodynamic responses to central hypovolemia, some responses to traumatic hemorrhage are not mimicked [13]. To this end, researchers have applied another model to simulate hemorrhage, blood withdrawal. In another study performed by our group, instantaneous amplitude modulations in heart rate and breathing rate frequency ranges were procured from PPG sensors from ear, finger and forehead locations from healthy volunteers who underwent 0.9 L of blood withdrawal [9]. These results indicated that the spectral amplitude of the PPG signal at the HR frequency range significantly decreased at different stages of volume loss, while the amplitude in the breathing rate frequency range did not change significantly [9].

The encouraging results from studies previously performed in our group [8] and [9] have led us to the idea of addressing the problem of blood loss detection in the intraoperative and trauma care settings. Therefore, the aim of this study was to automatically classify blood loss from PPG recordings acquired with portable, non-invasive POs developed in our lab [14] from trauma patients admitted to the UMass Medical Center as early as possible upon their arrival, and from healthy volunteers subjected to 0.9 L blood withdrawal using commercially available POs. We characterized every PPG recording with a feature vector that has, to the best of our knowledge, not been previously used by other researchers, consisting of four features: the goodness of fit (*r*^2^), the slope of the trend line, percentage change, and the absolute change between amplitude estimates in the heart rate frequency range at the first and last time points. Independently, every patient was assigned to one of two classes (Blood loss (BL)/No blood loss (NBL)) by adjudication of all available clinical information compiled during the data collection by UMass physicians. Subsequently, we performed automatic classification by applying the well-known support vector machines (SVM) algorithm, and obtained high overall accuracy, sensitivity and specificity.

## Materials and methods

### Experimental setup

Thirty six participants were enrolled in this study, of which twenty seven were trauma patients (age: 37 ± 11.3 years (mean ± SD), height: 174.5 ± 10.9 cm, weight: 89.9 ± 23.8 kg, 21 males) admitted to the UMass Medical Center, while the remaining nine were healthy male volunteers (age: 28 ± 2.9 years (mean ± SD), height: 174.6 ± 5.2 cm, weight: 80.1 ± 8.9 kg) subjected to blood withdrawal of 0.9 L as described in our prior paper [9]. All methods were carried out in accordance with relevant guidelines and regulations, while all experimental protocols were approved by the Institutional Review Boards of UMass Medical School, University of Connecticut, and Yale-New Haven Hospital. Prior to acquisition of any signals, an informed consent was obtained from all participants.

**Fig 1:**
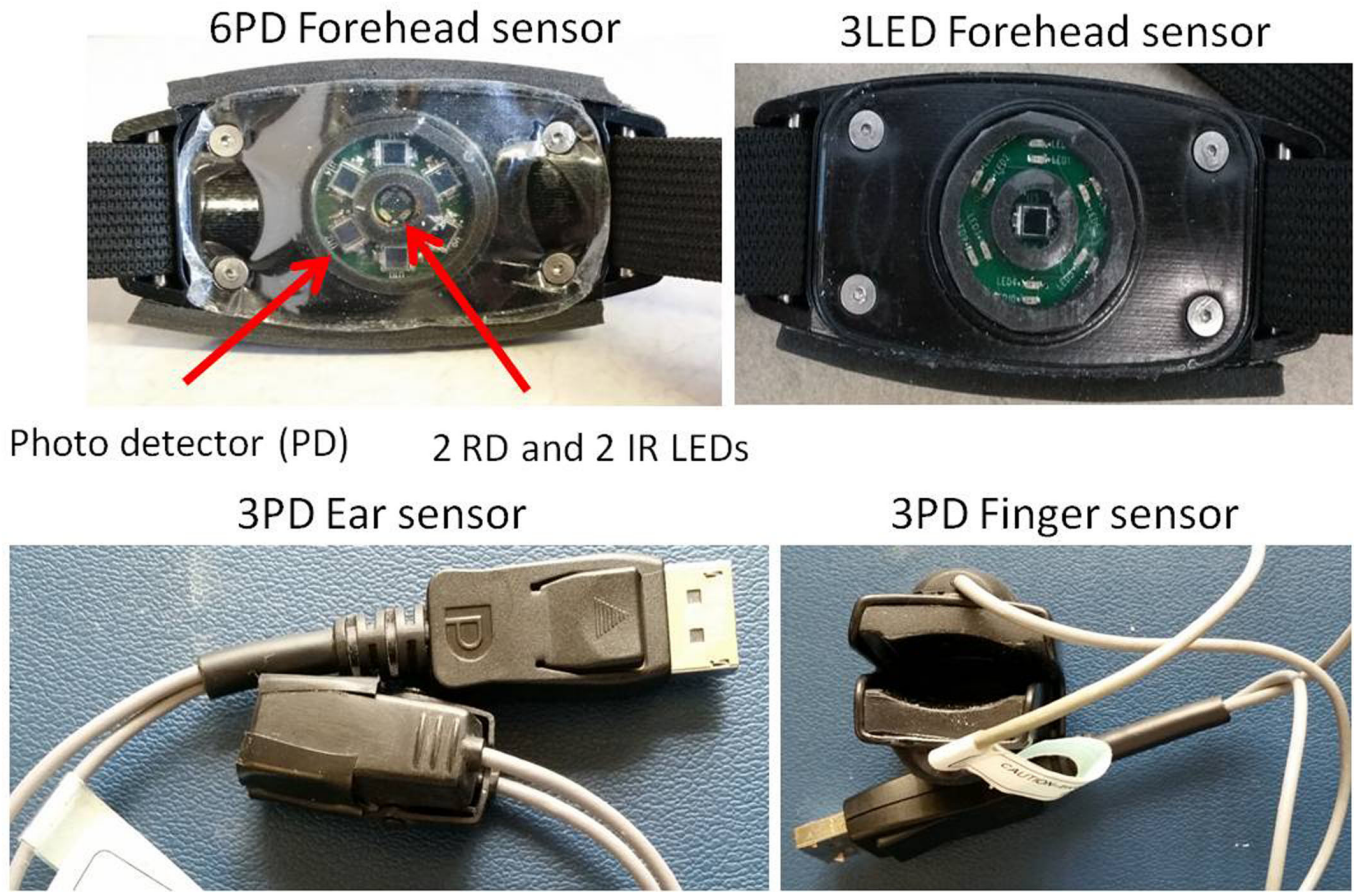
Multichannel POs.

Four types of non-invasive multichannel POs (MCPOs) that were developed in our lab [14] were used on trauma patients: 1) a 6PD (photo detector) forehead MCPO providing 6 PPG channels; 2) a 3LED (light emitting diode) forehead MCPO providing 3 PPG channels; 3) a 3PD ear MCPO; and 4) a 3PD finger MCPO, both providing 3 PPG channels. Note that the two forehead MCPOs were not used at the same time, and only three MCPOs were recording simultaneously on different locations on the skin. It is worth mentioning that in each of the MCPO sensors PPG signals were recorded simultaneously from different channels. The 6PD forehead, 3LED forehead, 3PD ear, and 3PD finger MCPOs are shown in Fig 1.

The sensors were placed on patients as early as possible upon their arrival. However, in some cases, data collection was delayed because of problems obtaining the patient's consent due to the severity of their condition. Also, we were unable to collect PPG data while patients were moved to computerized tomography (CT) scan. Note that some patients wore only one or two sensors at the same time due to the nature and severity of their injuries. For the experiments with healthy volunteers, subjects were equipped with three PPG probes (Modified Model 520A, Oxypleth®, Novametrix/Respironics, Wallingford, CT) that were placed also on forehead, ear and finger [9]. Overall, sixty seven recordings were obtained from trauma patients during blood loss of 0.01 to 2 L, estimated by UMass physicians, in the time span that varied from 20 to 158 minutes, while twenty seven were procured from healthy volunteers enrolled in our previous study during 0.9 L blood withdrawal that lasted between 12 to 40 minutes [9].

The blood loss in trauma patients was estimated by examining all available clinical data, while the American College of Surgeons (ACS) (Advanced Trauma Life Support) hemorrhage classification was used as a guide. The ACS specifies a major hemorrhage into Class I (mild) to Class IV (severe). The clinical data could include any of the following: nature of the injury, estimated pre-hospital blood loss, vital signs (e.g., heart rate and systolic blood pressure), vital sign changes over time, change in lab values over time (e.g., declining hemoglobin, lactic acidosis indicating poor perfusion due to blood loss), blood loss measured in the operating room, blood loss estimated by imaging tests (e.g., visible blood in the abdomen on CT scan), clinical notes indicating estimates of blood loss and when bleeding was controlled by medical care. UMass physicians examined all these available data over time and considered how treatment impacted blood loss over time. Two physicians independently reviewed the information and estimated blood loss by class and the amount. Disagreements were resolved by a third independent rater and a group consensus estimate was achieved. It is worth mentioning that among 67 recordings, forty eight were obtained from patients during blood loss and/or intervention and fluid resuscitation. Among those 48, twenty six recordings were obtained from seventeen actively bleeding patients. Note that 5 recordings of those 26 were acquired from three intubated trauma patients, collected in the operating room during emergency surgery.

Every MCPO collected multiple PPG recordings, depending on the number of PPG channels, and all were stored internally in the device and analyzed offline using MATLAB® (R2016a, The Mathworks, Inc., Natick, MA, USA). In addition, the annotation data (details regarding the patient’s medical condition and tables with intravenous (IV) fluid and blood intakes and outputs) were compiled during the entire data collection by the UMass physicians. At the end of data collection, every patient’s recording was assigned to one of the classes (BL/NBL) by adjudication of all available clinical information by 2 or 3 physicians. Note that physicians were blinded to the results of the analysis performed on PO data.

The summary of the used sensors and PPG recordings is provided in Table 1.

**Table 1:** The summary of sensors and PPG recordings used in the study.

Sensor/Location	Forehead		Ear	Finger	Total
**No. recordings (trauma patients)**	25 (6PD)	2 (3LED)	9	31	67
**No. recordings (healthy volunteers)**	9		9	9	27

### Data processing

**Fig 2:**
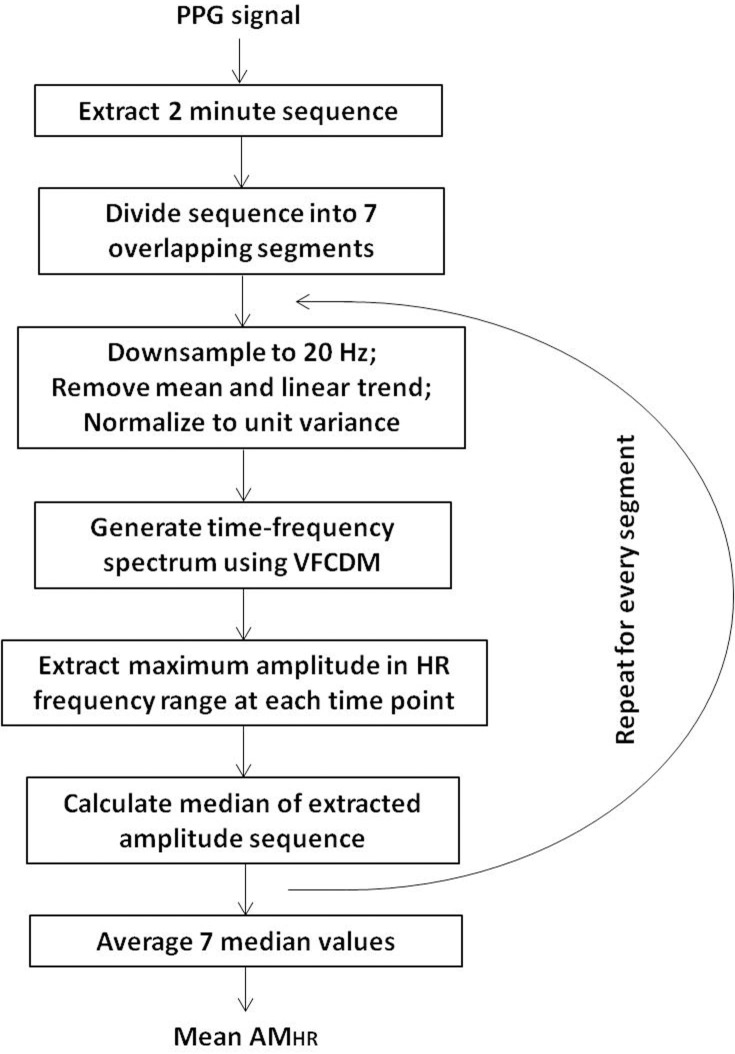
Processing steps for calculating the amplitude estimates in the HR frequency range.

The sampling frequency of all photoplethysmographic signals was 80 Hz. As was mentioned earlier, every sensor produces several PPG signals, therefore, prior to data processing we chose only one with the best signal-to-noise ratio. This was achieved by selecting the channel with the minimum Shannon entropy value. The PPG channel that resulted in a signal the least corrupted with motion artifacts or skin contact issues, or the signal with the most prominent amplitudes, was chosen for further data processing. Throughout the whole signal, we extracted 2 minute PPG sequences at various time instances, while within every sequence we shifted a 1 minute window in 10 second increments. The details of the data processing steps to estimate the amplitude within the HR frequency range (AM_HR_) from the extracted 2 minute sequences can be found in [8, 9, 11], while the summary of these steps is shown in Fig 2. Time-frequency spectra of every one minute segment were obtained using the variable frequency complex demodulation (VFCDM) algorithm, as was explained in detail in the references [8, 15, 16]. PPG signals obtained from people with symptoms of acute hemorrhage vary over time, especially the amplitude and frequency of heart rate, hence the application of the time-frequency analysis to these signals is a suitable tool that allows us to track these changes over time.

After AM_HR_ were calculated for various time instances, we plotted them against their corresponding time points and determined the trend of the data. The results of the trend analysis were presented by the goodness of fit (*r*^2^) and the slope of the trend line. If the slope of the line is positive, then we consider the person is euvolemic, and vice versa. This idea was successfully used in one of our previous papers [11]. In addition, we calculated the percentage change and the absolute change between AM_HR_ values at the first and last time points, as they serve as indicators of euvolemia if positive, and hypovolemia if negative. This rationale was based on the results presented in [6, 9], where the authors have shown that the spectral amplitudes at heart rate frequency range significantly decreased during the blood withdrawal. Hence, we proposed to use the feature vector consisting of those 4 features, as we hypothesize that their combination can give us better and more objective distinction between Blood loss and No blood loss classes.

**Fig 3:**
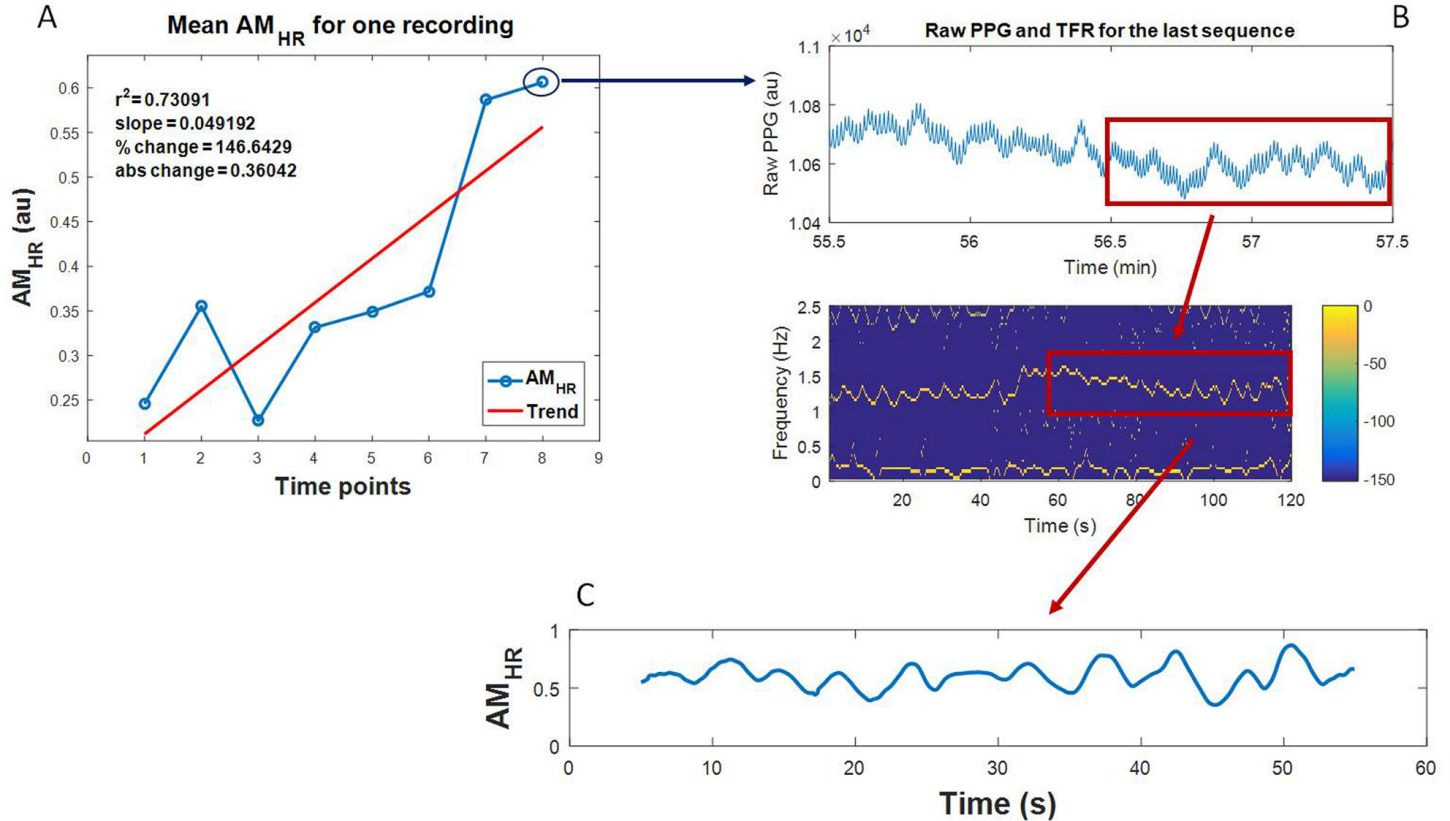
An illustrative example of a calculated feature vector for one trauma patient’s recording. **A**: The plot of mean AM_HR_ values at various time points, the trend line, and the corresponding features. **B**: The plot of the raw PPG and the corresponding VFCDM TFR of the 2 minute sequence at the last time point (point 8). **C**: Sequence of AM_HR_ values extracted from the last 1 minute TFR.

An illustrative example of a plot of estimated mean AM_HR_ values at eight time points for one trauma patient’s recording is depicted as the blue line in Fig 3A, while the corresponding trend line is represented as a red line. The calculated features are shown in the top left corner of the same graph. Note that during this data collection, the UMass physicians adjudicated the recording as NBL, as ongoing bleeding was minimal and a sufficient amount of IV fluids were received. The 2 minute sequence of raw PPG recording at the last time point (point 8 in Fig 3A) and its corresponding VFCDM time-frequency representation (TFR) are shown in Fig 3B. It is worth mentioning that a strong frequency component visible below 0.5 Hz in the Fig 3B corresponds to the breathing rate. The sequence of maximum amplitudes extracted from the heart rate frequency range (AM_HR_) of the last 1 minute segment of the time-frequency spectra (embodied within the red rectangle in Fig 3B) is depicted in Fig 3C.

### Dataset

In total, we collected 67 recordings from trauma patients with custom-made MCPO sensors, and 27 from healthy volunteers (enrolled in our previous study [9]) with commercially available POs on three locations: forehead, ear and finger. Every recording was characterized by four features: *r*^2^, the slope of the trend line, percentage change and the absolute change between AM_HR_ values at the first and last time points. Note that although different POs were used for data acquisition, the applied data analysis was exactly the same.

Each recording can belong to only one of the two classes–BL and NBL. A reference adjudication was made by UMass physicians. Among all recordings from trauma patients, 65 were classified as NBL by the physicians, while two were labeled as BL. Most of the patients needed an immediate treatment from emergency medical responders even prior to their arrival due to the severity of their injuries, so they had already received some intravenous fluids and blood units, and had their hemorrhage under control by the end of data recording, which resulted in such a high number of NBL recordings.

The remaining 27 recordings were from healthy volunteers who were subjected to 0.9 L blood withdrawal and hence denoted as BL [9]. This means that there were 65 recordings with the NBL label, and 29 with the BL label. In order to have a fair and objective classification process and not to give more weight to the class that has more recordings, we permuted the recordings of the NBL class and randomly selected 29, so that two balanced classes were formed, with *N*_BL_ = *N*_NBL_ = 29. Therefore, our dataset consisted of *N* = 58 recordings, where each was described with 4 features. Since there are many ways to randomly select 29 recordings from 65, we permuted all recordings in the NBL class and randomly selected 29 of them ten times, and formed 10 distinct datasets, each of dimension 58x4. This way we are ensuring the objectiveness of the classification results.

**Fig 4:**
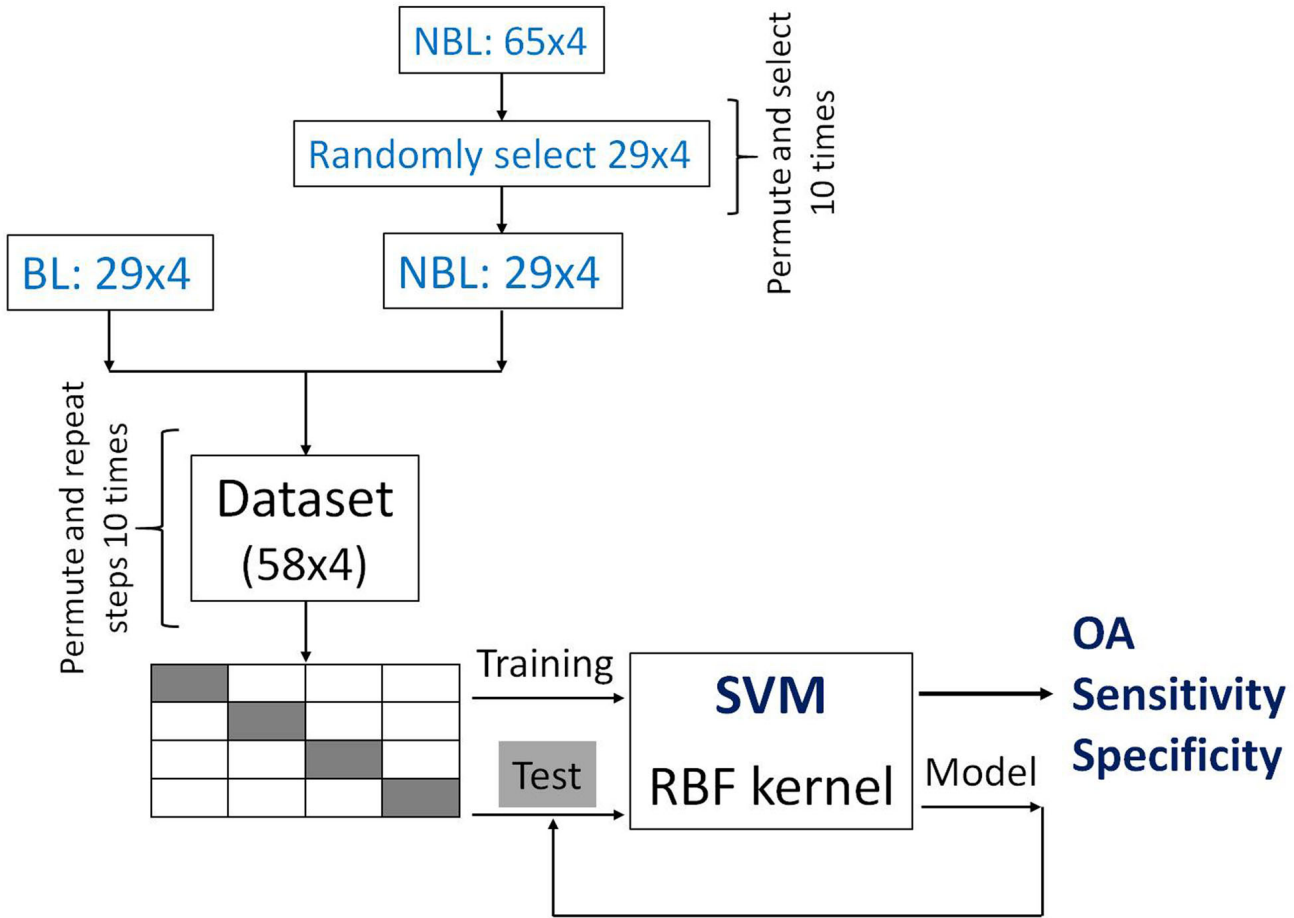
An illustration of the classification process.

The whole dataset was separated into training and test sets using 4-fold cross-validation, in order to obtain more reliable classification results. The order of appearance of recordings in each dataset can influence the selection of training and test sets during the 4-fold cross-validation process, and thus the outcome of the classification as well. To overcome this susceptibility, recordings and their corresponding features in every of the ten formed datasets were permuted 10 times as well. Therefore, we performed classification 100 times, and the final values of overall accuracy, sensitivity and specificity were obtained by averaging those procured in each run. The illustration of the classification process is shown in Fig 4.

The goal of this study was to perform automatic classification, that is, to use objective features to represent each recording so that the machine learning algorithm could distinguish between the BL and NBL classes as accurately as possible. To this end, we performed SVM with RBF kernel [17, 18] using LIBSVM toolbox v3.12 [19]. This type of SVM has two parameters, *γ* and *C*, and both were varied in order to achieve the optimal results. Parameter *γ* was varied in the following manner: 0.001, 0.005, 0.01, 0.05, 0.1, 0.15, 0.2, while regularization parameter *C* was varied as follows: 0.01, 1·10^*i*^, 2·10^*i*^, 5·10^*i*^, *i* = 0, 1,…, 18. In each of the 100 runs, we obtained classification results for every combination of the two parameters. Note that there are 7 distinct values for *γ* and 58 for *C*. At the end, classification results were averaged for every combination separately.

Moreover, we explored the ability of the proposed features and algorithm to distinguish between the same classes at an earlier stage, i.e., at the point when only about 0.45 L of blood had been withdrawn from our healthy volunteers. The same processing steps were applied to the same recordings acquired from healthy volunteers, but only to the portions up to about 0.45 L of blood withdrawal. This way, new datasets were formed with the same dimension (58x4) and the same order of appearance of recordings, but with different values of feature vectors.

## Results

Classification was performed one hundred times for every combination of the parameters γ and *C*, to prevent the order of appearance of recordings to influence and bias the outcomes. Obtained values of overall accuracy, sensitivity and specificity were averaged to procure the final results. The highest OA was 88.38%, while sensitivity and specificity were 88.86% and 87.90%, respectively, when parameters of the SVM classifier were: *γ* = 0.001 and *C* = 2, and are shown in Table 2 in bold. These results indicate that by representing each recording with the selected 4 features we are able to perform automatic discrimination between hypovolemia and euvolemia among trauma patients in the intraoperative setting and healthy volunteers with high accuracy, sensitivity and specificity, despite the fact that the PO sensors used on these two independent groups of participants were not the same. Therefore, our algorithm and choice of features are suitable for the task at hand. It is worth mentioning that our false positive rate was greater than our false negative rate, which is a promising result and coincides with the observation provided by Kneale [20], that it is thought better to err on the side of caution since the risk of death from hemorrhage due to lack of intravenous resuscitation is greater than the one from fluid overload.

**Table 2:** Confusion matrix (sensitivity and specificity are in bold) and overall accuracy for the optimal parameters of SVM with RBF kernel for 58 recordings from trauma patients and healthy volunteers subjected to 0.9 L blood withdrawal.

	True BL	True NBL
**Predicted BL**	**88.86%**	12.10%
**Predicted NBL**	11.14%	**87.90%**
**OA**	**88.38%**	

In addition, automatic distinction between the two classes when about 0.45 L of blood had been withdrawn from our healthy volunteers was performed. The time span from the start of blood withdrawal until about 0.45 L of blood was withdrawn varied from 7 to 35 minutes. The overall accuracy, sensitivity and specificity are presented in Table 3. The results indicate that the distinction between hypovolemia and euvolemia among two separate groups of participants is possible even at an early stage of hemorrhage.

**Table 3:** Confusion matrix (sensitivity and specificity are in bold) and overall accuracy for the optimal parameters of SVM with RBF kernel for 58 recordings from trauma patients and healthy volunteers subjected to about 0.45 L blood withdrawal.

	True BL	True NBL
**Predicted BL**	**69.62%**	24.56%
**Predicted NBL**	30.38%	**75.44%**
**OA**	**71.74%**	

## Discussion

Accurate detection of blood loss is popular topic in the literature. There are two frequently used models to simulate blood loss: LBNP and blood withdrawal. It is worth mentioning that it is difficult to compare results from our study to the results obtained in LBNP studies since the conditions and the form of reported results are different due to the fact that some responses to traumatic hemorrhage are not mimicked in LBNP models [13].

In a study previously performed in our lab, healthy volunteers were subjected to 0.9 L blood withdrawal, while PPG signals were recorded from ear, finger and forehead locations [9]. Similarly as in Selvaraj and co-workers [8], the time-varying spectral amplitude of PPG waveforms in the heart rate and breathing rate frequency ranges were obtained as features, where spectral amplitudes at HR frequency significantly decreased [9]. We cannot perform straight-forward comparison with the results reported in [9], since sensitivities and specificities were reported for every sensor separately, while we report the sensitivity and specificity for all sensors together. Stewart and co-workers estimated the compensatory reserve index from PPG signals to detect blood loss in healthy volunteers subjected to blood withdrawal of 0.45 L [21]. Although the authors achieved sensitivity and specificity of 84% and 86%, respectively, the same group of subjects was used for representing both classes (Blood loss/No blood loss), which introduced a bias into the reported results [21]. Convertino and colleagues performed a controlled study where twenty healthy volunteers were subjected to blood withdrawal of about 1.2 L, and obtained sensitivity and specificity of 80% and 75.9%, respectively [22], compared to our study where higher values of both sensitivity and specificity, 88.86% and 87.9% respectively, on a larger pool of subjects were achieved, both in intraoperative and controlled settings. Moreover, the technique proposed by Convertino's lab [21, 22] requires the baseline values, whereas our technique and features could detect blood loss even when data collection was initiated after blood loss had begun. A Nexfin device (non-invasive arterial blood pressure monitor) was used for detection of blood loss in spontaneously breathing subjects undergoing blood donation of 0.5 L using hemodynamic parameters, where pulse pressure variation and systemic vascular resistance index increased, as opposed to the cardiac index [23]. Due to the different forms of reported findings, a direct comparison of our results with the results reported in related studies (except for [21, 22]) is not very easy to perform.

To the best of our knowledge, there are just a few studies using data acquired outside of controlled setting. Cooke and co-workers have performed retrospective analyses of pre-hospital trauma patient records, both survivors and non-survivors, and determined that the high frequency to low frequency ratio derived from heart period (rate) variability analysis was able to distinguish between these two groups and was higher for non-survivors [24]. However, the efficacy of the study relies on clean ECG signals recorded in helicopters and an accurate peak detection algorithm, which can be a very limiting factor for broader applications, for instance in combat casualty care. Mackenzie et al showed that by adding features obtained from PPG signal to the single value of HR improved prediction of blood transfusion needs [10]. Again, the straight-forward comparison of our results to results reported in [24] and [10] cannot be easily performed due to different study setups and forms of reported results. Liu et al investigated the diagnostic performance of a platform for analysis of four vital signs (HR, respiratory rate, systolic BP, and pulse pressure) and their combinations en route to trauma center [25]. The authors applied a set of multivariate regression models to vital signs and their combinations, and averaged their outputs to obtain a single output value to obtain the sensitivity of 76% for 24 hr packed red blood cells (PRBC) of 9 or more units, and specificity of 87% for 24 hr PRBC of 0 units [25]. Note that we have obtained significantly higher sensitivity (88.86%) and slightly higher specificity (87.90%) with the proposed set of 4 features and SVM algorithm. In our recent study, where application of a similar technique was performed on PPG recordings obtained during trauma care, euvolemia was detected with an accuracy of about 79% [11]. Now, with a higher enrollment rate, we were able to achieve a higher overall accuracy of 88.38%. Note that due to the fact that all trauma patients were in an emergency situation and that the FDA rules required us to obtain the informed consent by the trauma patients or their proxy prior to beginning data collection, most of the patients had their hemorrhage under control by the time of data recording. Ideally, our goal is to obtain PPG recordings from subjects prior to, during, and after blood loss. That way, we may be able to track the changes in the AM_HR_ on an everyday basis in non-controlled setting, which may help us to better understand and improve the detection of hemorrhage.

The goal in all of the aforementioned studies was to propose a feature vector that could provide early detection of blood loss, since traditional vital signs show abrupt changes only at the critical level, and the reported results are very promising. However, the main limitation was that they were performed in well-controlled settings, where their translation to critically ill patients and intraoperative settings is not easy. There are, however, exceptions in the literature [11, 24, 25]. After obtaining promising results by using only the trend (slope) of the AM_HR_ values over time from trauma patients admitted to the hospital [11], here we added the goodness of fit, the percentage change and the absolute change between estimated AM_HR_ values at the first and last time points as characteristics of PPG recordings, and obtained high accuracy, sensitivity and specificity.

The ultimate target population of our study are the trauma patients and battlefield soldiers. Unfortunately, we could not enroll any battlefield soldiers due to technical limitations. As a result, we enrolled the next closest population to battlefield soldiers–trauma patients (people who were in car accidents, who had gun and/or knife wounds, who had internal bleedings due to falling down, etc.) arriving at the UMass Emergency Department with suspected hemorrhage. The estimated blood losses ranged from 0.01 to 2 L. Due to the severity of their injuries, these patients received the first treatment from the emergency medical responders, right after the accident and before any of the sensors could be placed on them. In addition, during the data collection, even though some patients were still actively bleeding, they were receiving necessary blood units and intravenous fluids in order to compensate for the blood lost. By the end of the data recording, due to all interventions, all except 2 patients had their hemorrhage under control, and physicians labeled them as NBL. It is worth mentioning that 3 trauma patients with 5 PPG recordings were in the operating room with ongoing bleeding during the data collection, which makes our technique applicable for intraoperative and battlefield settings. However, these patients were labeled as NBL by physicians, as the hemorrhage was surgically controlled towards the end of recording and all received more IV fluids and blood products than estimated blood lost.

Currently, the selection of the one PPG channel to be processed is manual. However, as part of our future work, we intend to automate this step by using algorithms and metrics previously developed in our lab [26–30].

We believe that our results could be a good initial point for the possible future study on battlefield soldiers, where the same data processing technique, same features and algorithm could be applied on PPG recordings. In that case we foresee that soldiers would wear portable miniaturized pulse oximeters at all times, while medics would remotely monitor PPG signals and react immediately in case blood loss is detected.

## Conclusions

We proposed a set of PPG features and algorithm for an automatic distinction between BL and NBL classes. We analyzed PPG signals acquired with portable, non-invasive POs developed in our lab from trauma patients in the intraoperative setting and trauma care, and we collected PPG signals with commercially available POs from healthy volunteers subjected to blood withdrawal of 0.9 L. We proposed the use of four features based on the estimated amplitude values in the heart rate frequency range and trend analysis to perform automatic classification of hypovolemia using support vector machines. The overall accuracy was 88.38%, sensitivity 88.86% and specificity 87.9%. It is worth noting that the proposed technique for blood loss detection does not require baseline values. Our features and algorithm show robustness, since they can be applied to both custom-made and commercial PO devices, as well as to different groups of participants, in both controlled and clinical settings, including intraoperative settings. Note that although the data were collected with different devices, the same data processing techniques were applied. In addition, the proposed features and algorithm performed well even when moderate blood volume was withdrawn. Motion artifacts and/or changes in the contact between the sensor and skin can disrupt PPG recordings, thus negatively impacting the data analysis and feature values. Furthermore, the classification process and detection of blood loss will produce unreliable results. By including the algorithm for motion noise artifacts developed in our lab [26–30], the proposed algorithm can be embedded into medical monitoring devices, and may improve blood loss detection in the presence of motion artifacts, which in turn may be beneficial and applicable in both intraoperative/emergency and combat casualty care.

In summary, we have proposed a novel set of four features: the goodness of fit (*r*^2^), the slope of the trend line, the percentage change and the absolute change between estimated amplitude values in the heart rate frequency range at the first and last time points, for automatic classification of blood loss. Moreover, the proposed features were obtained by applying the same data processing technique and high-resolution time-frequency algorithm to two independent groups of participants, trauma patients and healthy subjects, where PPG signals were obtained from different sets of pulse oximeters from up to three locations: ear, finger and forehead. In addition, PPG signals from trauma patients were acquired with portable non-invasive miniature multichannel pulse oximeters developed in our lab. High values of classification accuracy, sensitivity and specificity indicate the ability of the proposed method to be used in non-controlled setting (trauma care center) as well. It is worth noting that the proposed technique does not require baseline values, and that the accurate detection of blood loss could be procured even when data collection was initiated after blood loss had begun, which makes our technique superior compared to techniques proposed by other researchers.
